# Antifungal Activity of *Acorus calamus* Essential Oil Against Rice Blast Fungus *Magnaporthe oryzae* and Its Composition Characterization

**DOI:** 10.3390/plants15020332

**Published:** 2026-01-22

**Authors:** Shuzhen Deng, Ziyi Wang, Yusi Li, Yiming Liu, Zhiyi Kong, Ge Meng, Saige Jin, Anqi Zeng, Huan Liu, Shengming Liu

**Affiliations:** 1 College of Horticulture and Plant Protection, Henan University of Science and Technology, Luoyang 471023, China; wangziyi202027@163.com (Z.W.); lys18003801854@163.com (Y.L.); yimingliu2024@163.com (Y.L.); kong08092024@163.com (Z.K.); 15236100392@163.com (G.M.); 15896559865@163.com (S.J.); 13721639037@163.com (A.Z.); liuhuan@haust.edu.cn (H.L.); 2 Henan Province Engineering Technology Research Center of Green Plant Protection, Luoyang 471023, China

**Keywords:** *Acorus calamus*, essential oil, *Magnaporthe oryzae*, antifungal activity

## Abstract

Rice blast, caused by the fungal pathogen *Magnaporthe oryzae*, is one of the most devastating diseases affecting global rice production. Plant essential oils (EOs) have been considered as a promising green alternative to synthetic fungicides. In this study, the antifungal activities of five plant EOs—*Acorus calamus*, *Citrus reticulata*, *Syzygium aromaticum*, *Paeonia suffruticosa*, and *Melaleuca viridiflora*—against *M. oryzae* were evaluated using the mycelial growth rate method. Among them, *A. calamus* EO (ACEO) exhibited the most pronounced inhibitory effect, with an EC_50_ value of 0.37 μL/mL. It significantly delayed or inhibited conidial germination and appressorium formation. At higher concentrations (≥1 μL/mL), it also caused morphological abnormalities in appressoria. Observations by scanning electron microscopy (SEM) and transmission electron microscopy (TEM) revealed that the EO treatment caused hyphal surface wrinkling, cell wall thinning, organelle dissolution, and vacuolation. Pathogenicity tests further confirmed that ACEO reduced the virulence of the fungus remarkably, with nearly complete loss of pathogenicity at a concentration of 1 μL/mL. Finally, ACEO was analyzed using gas chromatography-mass spectrometry (GC-MS). The most abundant constituents identified were *β*-asarone (19.83%) and isoshyobunone (14.92%). Together, these findings demonstrate that ACEO impairs fungal pathogenicity by disrupting hyphal morphology and cellular integrity, highlighting its potential as an effective and eco-friendly fungicide for controlling rice blast.

## 1. Introduction

Rice blast, caused by the fungal pathogen *Magnaporthe oryzae*, is often referred to as the “cancer of rice” due to its devastating impact. This disease leads to global rice annual yield losses of 10% to 30%, equivalent to the annual food supply for approximately 60 million people [1,2,3]. It compromises both rice production and quality severely, posing a serious threat to global food security. Currently, the cultivation of resistant varieties is regarded as the most economical and effective strategy for controlling rice blast. However, the widespread cultivation of a single resistant variety, and the high diversity and genetic complexity of *M. oryzae* races often result in the rapid breakdown of resistance [4]. Moreover, the process of developing new resistant cultivars is time-consuming. Alternatively, the application of chemical fungicides remains the primary measure for controlling rice blast. Commonly used agents include sterol demethylation inhibitors, melanin biosynthesis inhibitors, and mitochondrial respiration inhibitors [5,6,7]. These chemical agents have played a crucial role in reducing the damage caused by *M. oryzae*. Nevertheless, excessive and improper use of these chemicals has led to the emergence of fungicide resistance, environmental pollution and ecological diversity concerns [8,9,10,11]. Therefore, there is an urgent need for the development of novel, highly efficient, low-toxicity, and environmentally friendly compounds for the control of rice blast.

Plant essential oils (EOs), which are secondary metabolites extracted from plant tissues, represent a valuable class of natural resources [12]. Currently, plant EOs are widely used in antimicrobial applications [13,14], food preservation [15], pest control [16], medicine [17], and other fields. These oils exhibit broad-spectrum biological activities, such as antimicrobial, antiviral, and antioxidant properties. Considering their natural origin, environmental friendliness, and multi-target mechanisms of action, plant EOs show huge potential in the fields of antimicrobial application and food preservation. In particular, for plant disease control, EOs have emerged as a promising and eco-friendly alternative to counteract the limitations and reduce the harmful impact of synthetic fungicides during the control of fungal plant diseases [18]. Notably, plant-derived EOs and their bioactive components have shown significant inhibitory effects in vitro against various phytopathogenic fungi, including *Botrytis cinerea* [19], *Penicillium expansum* [20], *Fusarium* spp. [21], *Alternaria* spp. [22], and *Colletotrichum* spp. [23]. For instance, *Citrus reticulata* EOs reduced the mycelial growth of *B. cinerea* by 54% after 96 h and increased the hyphae damage by 40%. Kou et al. reported that *Artemisia capillaris* EO showed a strong inhibitory effect on two *Alternaria* species, with a minimal inhibitory concentration (MIC) value equivalent to 5.0 μL/mL.

*Acorus calamus* (L.), a perennial aromatic medicinal plant of the Acoraceae family, is known for its pharmaceutical and medicinal value [24,25]. *A. calamus* EO (ACEO) has been demonstrated to possess insecticidal activity against various insect species, including *Spodoptera litura* [26], *Sitophilus zeamais* [27], and *Liposcelis bostrychophila* Badonnel [28]. Furthermore, studies indicate that ACEO exhibits significant antibacterial activity against *Pseudomonas aeruginosa* and *Staphylococcus aureus*, highlighting its potential as a natural preservative and antimicrobial agent [29]. However, research on the antifungal activity of ACEO against *M. oryzae* remains limited.

In this study, the antifungal activities of five plant EOs—*Acorus calamus*, *Citrus reticulata*, *Syzygium aromaticum*, *Paeonia suffruticosa*, and *Melaleuca viridiflora*—against *M. oryzae* were evaluated using the mycelial growth rate method. Among them, ACEO exhibited the best antifungal activity. We determined its impact on conidial germination and appressorium formation of *M. oryzae*. Furthermore, alterations in the ultrastructure of the fungal mycelia treated with ACEO were observed using scanning electron microscopy (SEM) and transmission electron microscopy (TEM). Finally, the effect of ACEO on the pathogenicity of *M. oryzae* was assessed, and its chemical composition was further analyzed by gas chromatography-mass spectrometry (GC-MS). This research aims to provide technical support for the development of efficient, safe, and long-lasting fungicidal agents to control rice blast.

## 2. Results

### 2.1. Primary Screening of the Selected Essential Oil

**Figure 1:**
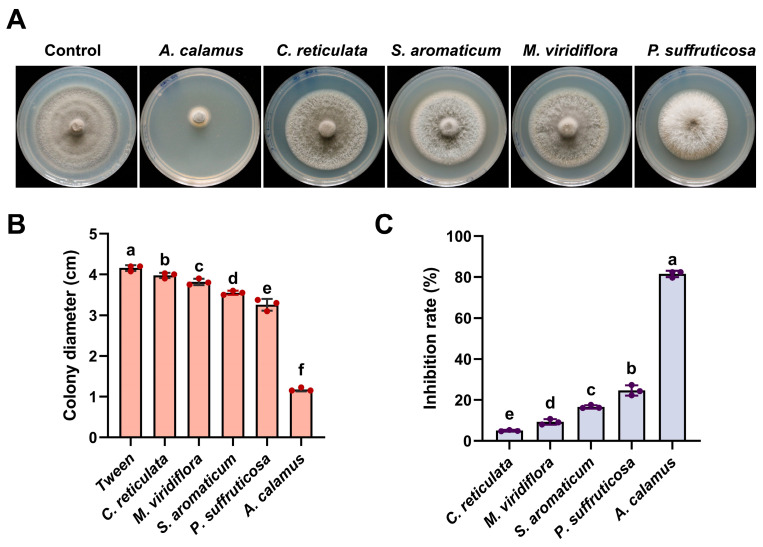
Effects of different EOs on the mycelial growth of *Magnaporthe oryzae*. (**A**) Morphology of colonies from the wild-type strain Guy11 cultured on CM medium supplemented with 0.1 μL/mL of the EO at 25 °C. Photographs were taken at 7 days post inoculation; (**B**) Bar chart showing the colony diameters of *M. oryzae* on plates treated with different EOs; (**C**) Bar chart showing the inhibition rates of different EOs. Means and standard deviations were calculated based on three independent experiments. Different lowercase letters above bars indicate significant differences (ANOVA, *p* < 0.05).

Based on the concentration gradient tests from preliminary drug screening, the test concentration of EO was set at 1.0 μL/mL. The antifungal activities of five EOs—derived from *Acorus calamus*, *Citrus reticulata*, *Syzygium aromaticum*, *Paeonia suffruticosa*, and *Melaleuca viridiflora*—against *M. oryzae* were evaluated using the mycelial growth rate method. As presented in Figure 1A–C, at 1.0 μL/mL, *C. reticulata*, *S. aromaticum*, *P. suffruticosa,* and *M. viridiflora* EOs showed moderate inhibitory effects on mycelial growth. In contrast, ACEO exhibited the most significant inhibition, with a mycelial growth inhibition rate of 81.56%.

### 2.2. The Inhibition of ACEO on Mycelial Growth of M. oryzae

**Figure 2:**
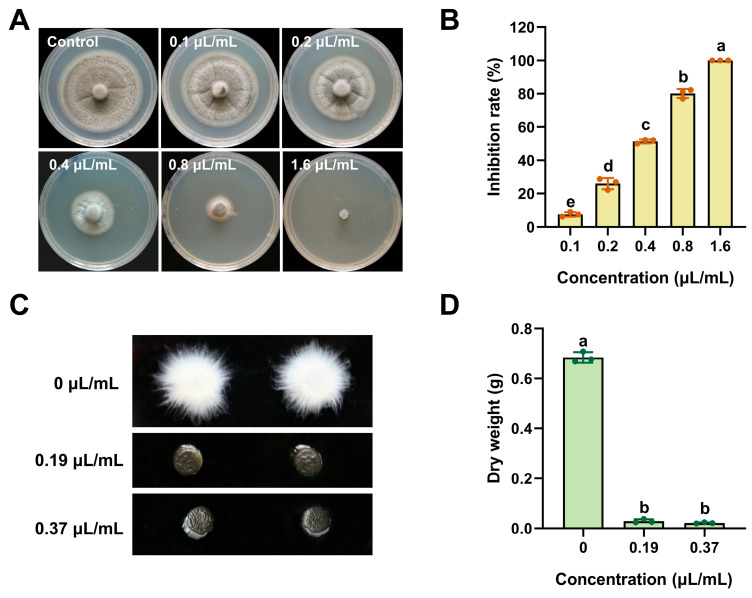
Inhibitory effects of different concentrations of ACEO on the mycelial growth of *M. oryzae*. (**A**) Morphology of colonies from Guy11 cultured on CM medium supplemented with different concentrations of ACEO at 25 °C. Photographs were taken at 7 days post inoculation; (**B**) Bar chart showing the inhibition rates of different concentrations of ACEO. (**C**) Mycelial morphology was examined and photographed after cultivation for 2 days at 28 °C in liquid CM medium with a gradient of thymol concentrations; (**D**) Bar chart showing dry weight of *M. oryzae* on plates treated with different concentrations of ACEO; Means and standard deviations were calculated based on three independent experiments. Different lowercase letters above bars indicate significant differences (ANOVA, *p* < 0.05).

ACEO, which has shown the highest antifungal activity in preliminary screening, was selected for median effective concentration (EC_50_) determination. As shown in Figure 2A,B, the mycelial growth of *M. oryzae* was inhibited by ACEO in a dose-dependent manner, with the EC_50_ value of 0.37 μL/mL. Subsequently, *M. oryzae* was inoculated into liquid CM medium supplemented with graded concentrations of the EO and shaken at 28 °C for 48 h. The control group developed relatively large and well-dispersed mycelial balls. In contrast, mycelial growth was markedly suppressed in treatments with 1/2 × EC_50_ and EC_50_ concentrations (Figure 2C). Mycelia from each group were then harvested, oven-dried, and subjected to dry weight measurement. The dry weight of mycelia in both treatment groups was significantly lower compared to the control (Figure 2D). Taken together, these results indicate that ACEO strongly inhibits the mycelial growth of *M. oryzae*.

### 2.3. The Inhibition of ACEO on Conidial Germination and Appressorium Formation of M. oryzae

**Figure 3:**
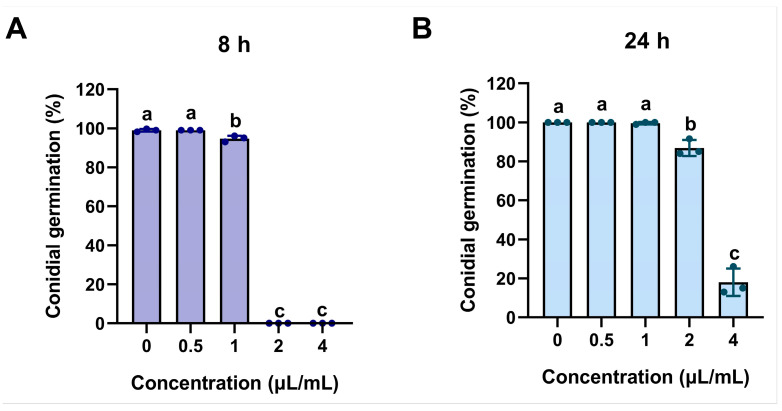
Inhibition effect of ACEO on conidial germination of *M. oryzae*. Bar chart showing the conidial germination rates at 8 (**A**) and 24 h post inoculation (hpi) (**B**). Different lowercase letters above bars indicate significant differences (ANOVA, *p* < 0.05).

To clarify the effect of ACEO on the conidial germination of *M. oryzae*, the conidial germination rates under treatments with different concentrations of the EO were determined. The results showed (Figure 3A) that after 8 h of incubation, the conidial germination rates in the control group, 0.5 μL/mL, and 1.0 μL/mL treatment groups all reached 100%, whereas the 2.0 μL/mL and 4.0 μL/mL treatments completely inhibited conidial germination. After extending the incubation time to 24 h, the germination rate in the 2.0 μL/mL treatment group recovered to approximately 90%, while that in the 4.0 μL/mL treatment group remained at a low level (Figure 3B). These results indicate that ACEO significantly inhibits the conidial germination of *M. oryzae* in a concentration-dependent manner.

**Figure 4:**
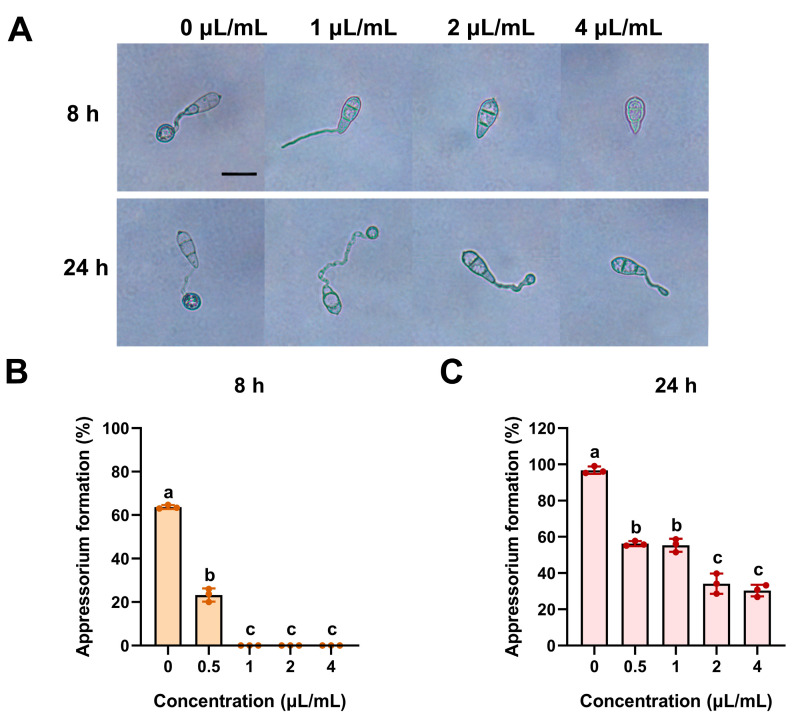
Inhibition effect of ACEO on appressorium formation of *M. oryzae*. (**A**) Conidial suspensions (1 × 10^5^/mL) of the wild-type strain Guy11 were spotted onto hydrophobic glass slides. Appressorium formation rates were assessed and photographed under a microscope after 8 h and 24 h. (**B**) Bar chart showing the appressorium formation rates at 8 hpi; (**C**) Bar chart showing the appressorium formation rates at 24 hpi. Different lowercase letters above bars indicate significant differences (ANOVA, *p* < 0.05).

The effect of ACEO on appressorium formation was further examined, and a similar concentration-dependent effect was observed (Figure 4). After 8 h of incubation, the appressorium formation rate in the 0.5 μL/mL treatment group was approximately 23%, which was significantly lower than that of the control group, whereas treatments with 1 μL/mL and higher concentrations completely inhibited appressorium formation (Figure 4A,B). At 24 h of incubation, the appressorium formation rates in the 0.5 μL/mL and 1.0 μL/mL treatment groups recovered to approximately 55%, while those in the 2.0 μL/mL and 4.0 μL/mL groups only recovered to about 30% (Figure 4C). Moreover, in treatment groups with concentrations ≥1 μL/mL, the formed appressoria showed abnormal morphology, mostly appearing flattened and oval-shaped (Figure 4A). These results indicate that ACEO not only significantly suppresses appressorium formation in *M. oryzae* but also induces morphological deformities in appressoria at high concentrations (≥1 μL/mL).

### 2.4. Effects of ACEO on M. oryzae Morphology

**Figure 5:**
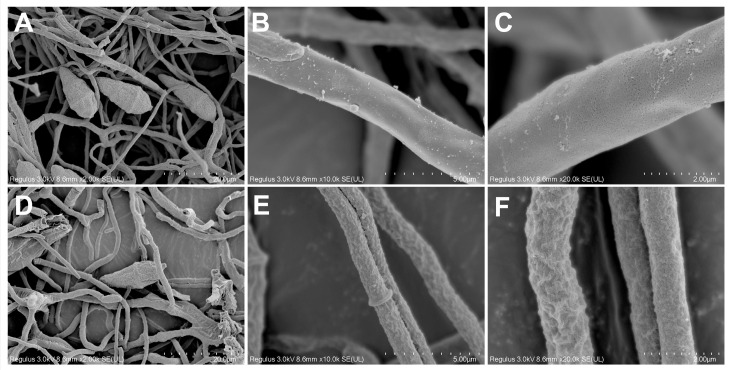
Scanning electron microscopy images of *M. oryzae* with or without ACEO treatment. (**A**–**C**) Control group; (**D**–**F**) Treated at EC_50_.

Scanning electron microscopy (SEM) was employed to examine the ultrastructural morphology of *M. oryzae* hyphae following treatment with ACEO, as shown in Figure 5. In the control group (Figure 5A–C), the hyphae exhibited an intact morphology with a smooth surface and uniform diameter. In contrast, after treatment with the EO, the hyphae displayed obvious fragmentation and significant surface deformation, including shrinkage, collapse, and localized rupture (Figure 5D–F). These results indicate that ACEO can disrupt the morphological integrity of *M. oryzae* hyphae.

**Figure 6:**
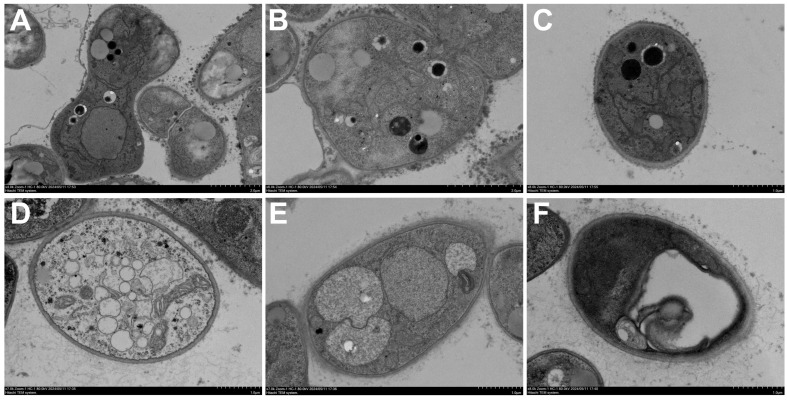
Transmission electron microscopy images of *M. oryzae* with or without ACEO treatment. (**A**–**C**) Control group; (**D**–**F**) Treated at EC_50_.

Furthermore, transmission electron microscopy (TEM) was used to observe ultrastructural changes in the hyphal cells (Figure 6). In the control group, the hyphal cells showed a clear structure: the cell wall was uniformly thick and closely attached to the plasma membrane, and organelles were evenly distributed with intact structures within the cytoplasm (Figure 6A–C). Conversely, in the EO-treated group (Figure 6D–F), the ultrastructure of the hyphae was severely disrupted. Specific alterations included: localized lysis of the cell wall and invagination of the plasma membrane; the appearance of numerous vacuolated structures in the cytoplasm; organelles appearing blurred, degraded, or even completely absent; along with evident cellular swelling. These findings demonstrate that ACEO significantly damages the ultrastructure of *M. oryzae* cells, affecting the normal morphology and distribution of organelles.

### 2.5. Effects of ACEO on M. oryzae Pathogenicity

**Figure 7:**
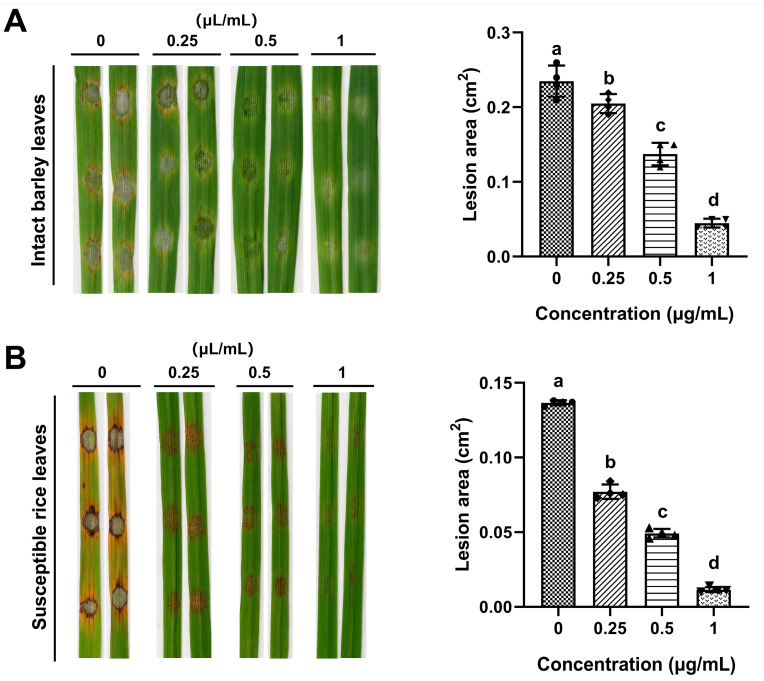
Effect of ACEO on the pathogenicity of *M. oryzae*. (**A**,**B**) Barley or rice segments were droplet-inoculated with conidial suspensions supplemented with different concentrations of ACEO. Bar chart showing the lesion areas in barley (**A**) and rice (**B**) leaves. Mean areas were calculated from six biologically independent samples at 5 days post-infection. Different lowercase letters above bars indicate significant differences (ANOVA, *p* < 0.05).

To investigate whether ACEO influences the infection process of *M. oryzae*, pathogenicity assays were conducted on both barley and rice plants. Detached leaves from 7-day-old barley or 2-week-old rice seedlings were inoculated with conidial suspension of *M. oryzae* (1–2 × 10^5^ conidia/mL) and maintained in a humid chamber. After 5 days of incubation, disease progression was evaluated. As shown in Figure 7, in the control group, typical spindle-shaped lesions with yellow halos developed on barley and rice leaves. As the concentration of ACEO increased, the conidia of *M. oryzae* gradually lost pathogenicity, and when the concentration of ACEO reached 1 μL/mL, conidia were nearly nonpathogenic to both rice and barley. These findings indicate that ACEO significantly attenuates the pathogenicity of *M. oryzae* in barley and rice in a concentration-dependent manner.

### 2.6. Chromatography-Mass Spectrometry (GC-MS) Analysis

The chemical constituents of ACEO were characterized using the GC-MS technique, and their relative contents (%) of major compounds in ACEO are presented in Table 1. ACEO was predominantly composed of *β*-asarone (19.83%) and isoshyobunone (14.92%). Other major constituents included zierone (8.30%), epishyobunone (8.07%), *α*-calacorene (4.81%), 6-epishyobunone (4.62%), *δ*-cadinene (4.52%), *α*-asarone (4.03%), isocalamenediol (2.99%), dehydroxy-isocalamendiol (2.98%), ledene (2.78%), and 4-trans-propenylveratrole (2.06%). These components made up around 79.91% of the content of the ACEO.

## 3. Discussion

Rice blast, caused by *M. oryzae*, is a devastating global disease that severely threatens rice production and food security [1,2]. Due to the issues of resistance and environmental contamination faced by traditional chemical fungicides, it is urgent to find novel, green, and efficient agents against *M. oryzae*. Botanical fungicides, characterized by their low toxicity, minimal residue, and reduced risk of resistance development, represent a promising direction in crop protection [30]. Among them, plant EOs, secondary metabolites extracted from plant tissues, have shown broad-spectrum biological activities, including antimicrobial, antiviral, insecticidal, and antioxidant properties. Currently, EOs have emerged as a promising and eco-friendly alternative to synthetic fungicides in the management of plant fungal diseases. To date, several plant-EOs have been confirmed to effectively inhibit the growth of *M. oryzae*, such as green tea EO [31], thyme EO [32], soybean seeds EO [33], *Aglaia odorata* Lour EO [34]. For instance, Liu et al. found that thyme EO exhibited excellent antifungal efficacy against *M. oryzae*, with an EC_50_ value of 0.29 μL/mL. The most predominant constituent in thyme EO was thymol which is a well-studied antimicrobial compound. The mode of action of thymol involves the disruption of the melanin biosynthesis pathway, which has been widely recognized as a critical virulence pathway in plant pathogenic fungi [32].

*Acorus calamus* (L.) EO, derived from plants of the Acoraceae family, has been demonstrated to possess insecticidal and antimicrobial activity [28,29,35]. For instance, ACEO exhibited contact toxicity against *Liposcelis bostrychophila*, with an LD50 value of 100.21 µg/cm^2^ [28]. Al-Mijalli et al. reported that ACEO exhibited significant antibacterial activity against *Pseudomonas aeruginosa* and *Staphylococcus aureus* with inhibition zones of 15.58 ± 0.68 mm and 20.11 ± 0.28 mm, respectively [29]. In addition, Phongpaichit et al. reported promising antifungal activity of *A. calamus* extracts against *Microsporum gypseum*, *Trichophyton rubrum*, and *Penicillium marneffei*, with IC_50_ values of 0.2, 0.2, and 0.4 mg/mL, respectively. However, these reports primarily focus on insects, bacteria, or fungi relevant to human health. Research on its activity against plant pathogenic fungi, particularly *M. oryzae*, remains limited and unclear.

In this study, we evaluated the antifungal activity of five plant EOs against *M. oryzae*. Among them, ACEO demonstrated the most potent inhibitory effect, with an EC_50_ value of 0.37 µL/mL. This efficacy is comparable to that reported for thymol EO (0.29 µL/mL) in recent literature, but is notably higher than that of green tea EO, which showed only 38.88 ± 0.3% inhibition at a volume concentration of 1000 µL/Petriplate [31,32]. Notably, ACEO significantly interfered with key pathogenic developmental stages of *M. oryzae*, including conidial germination and appressorium formation. At concentrations ≥ 1.0 μL/mL, ACEO not only suppressed conidial germination but also completely inhibited normal appressorium development and induced morphological abnormalities, such as flattened and oval-shaped appressoria. The inhibition of these early developmental stages is of particular significance, as conidial germination and appressorium formation are critical for the initial infection and disease cycle of *M. oryzae* [36]. Although a partial recovery in conidial germination and appressorium formation was observed after 24 h at certain concentrations, the extent of recovery remained limited, especially at higher concentrations (4.0 μL/mL). This reversible inhibition suggests that ACEO may primarily exert a fungistatic rather than a fungicidal effect at low to intermediate concentrations, temporarily suppressing fungal development without causing immediate lethality. Phenotypic screening targeting these foundational pathogenic steps represents a validated strategy for the discovery of novel antifungal agents [37]. Thus, the pronounced activity of ACEO against these key pathogenic steps underscores its potential as a promising candidate for further development as an eco-friendly fungicide.

Ultrastructural analyses provided direct morphological evidence for the antifungal mechanism of ACEO against *M. oryzae*. Observations via scanning electron microscopy (SEM) revealed a pronounced collapse and shrinkage of the hyphal surface following treatment. Transmission electron microscopy (TEM) further demonstrated substantial subcellular disorganization, including marked thinning of the cell wall, disintegration of intracellular organelles, and the formation of extensive vacuoles. This pattern of cellular damage—characterized by compromised cell wall architecture, loss of plasma membrane integrity, and cytoplasmic organelle dissolution—aligns with the documented effects of other plant EOs on phytopathogenic fungi, such as *B. cinerea* [38] and *P. oxalicum* [39]. The antifungal mechanisms of EOs are frequently associated with the disruption of ergosterol and melanin biosynthesis, direct impairment of membrane integrity and the induction of reactive oxygen species (ROS) accumulation. It is plausible that AECO may interfere with one or several of these key pathways, thereby leading to the loss of membrane stability, weakening of cell wall integrity, and eventual organelle degradation as visualized via TEM and SEM. Future studies employing molecular and biochemical assays will be necessary to validate these hypotheses and clarify the primary targets of ACEO.

In the pathogenicity assay, ACEO exerted a concentration-dependent inhibition on the pathogenicity of *M. oryzae*. At a concentration of 1 μL/mL, the pathogenicity of the fungus on host leaves was nearly completely abolished. This finding is closely aligned with the previously observed inhibitory effects of ACEO on conidial germination and appressorium formation. Moreover, the structural alterations observed at the ultrastructural level fundamentally disrupted cellular integrity, which ultimately led to a significant impairment of pathogenicity of *M. oryzae*. Finally, the chemical composition of ACEO was analyzed. According to the GC-MS analysis, the main components of ACEO were *β*-asarone (19.83%) and isoshyobunone (14.92%). Other compounds, present in smaller proportions, included zierone (8.30%), epishyobunone (8.07%), *α*-calacorene (4.81%), 6-epishyobunone (4.62%), *δ*-cadinene (4.52%), *α*-asarone (4.03%), isocalamenediol (2.99%), dehydroxy-isocalamendiol (2.98%), ledene (2.78%), and 4-trans-propenylveratrole (2.06%). This is consistent with other previously reported studies [40,41] that found *β*-asarone was the most significant compound. However, the content of any constituent of EO may differ depending on the cultivars, ripening stages, extraction techniques, and the tested plant organ [42].

In conclusion, this study demonstrates that ACEO exhibits significant inhibitory activity against *M. oryzae*, the causal agent of rice blast. The low EC_50_ value (0.37 µL/mL) for mycelial growth inhibition highlights its potent antifungal efficacy compared to other tested EOs. More importantly, ACEO significantly disrupted key pathogenic developmental stages of *M. oryzae*, including conidial germination and appressorium formation, and severely damaged hyphal ultrastructure, ultimately leading to a marked reduction in fungal virulence. These findings supported its potential as an eco-friendly candidate for disease management. Nevertheless, several practical challenges must be addressed before ACEO can be considered for agricultural use. Its phytotoxicity potential, high volatility, stability under field conditions, and formulation challenges represent important limitations that could affect real-world efficacy. Furthermore, the specific active constituents of ACEO and their synergistic mechanisms require further in-depth investigation. Future studies should focus on the identification of active constituents and molecular elucidation of action targets, thereby providing a novel theoretical foundation for the green and sustainable control of rice blast.

## 4. Materials and Methods

### 4.1. Materials and Reagents

*M. oryzae* strain Guy11 used in the present study was isolated from rice blast disease, provided by Zhejiang University. Standard growth and storage procedures for fungal strains were performed, as described previously [32]. Plant EOs used in this study were purchased from Luxury Bloom Biotech Co., Ltd. (Shanghai, China) and were maintained at 4 °C until utilization.

### 4.2. Mycelial Growth Assay

Mycelial growth was assessed by measurement of colony diameters in plate cultures. A stock solution of EO was prepared using 5% Tween-80. An appropriate amount of this stock solution was added to 10 mL of Complete Medium (CM) to prepare CM agar plates containing different concentrations of EO. A CM plate containing 0.1% Tween-80 served as the blank control. Mycelial plugs (5 mm in diameter) of *M. oryzae* were inoculated onto the center of both the plates containing different concentrations of EO and the blank control plates. Afterwards, all plates were placed in an incubator at 25 °C for 7 days. The colony diameter was measured using the cross-bracketing method, and the data were subjected to statistical analysis. The EC_50_ Value was calculated using SPSS 26.0 [43].

### 4.3. Dry Weight Assay

Dry weight was measured using the liquid shake-flask cultivation method. Equal amounts of mycelia were inoculated into CM liquid medium containing different concentrations of *A. calamus* EO, with an equal amount of Tween-80 as a blank control. The cultures were incubated at 28 °C and 180 rpm for 48 h, with three replicates for each treatment. The mycelia were cleaned with sterile water and then dried to a constant weight. The dry weight of the mycelia was measured using an analytical balance (precision: 0.1 mg), and the inhibition rate was calculated.

### 4.4. Conidial Germination and Appressorium Formation Assay

The stock solution of ACEO was diluted to prepare solutions at different concentration gradients. Conidia were collected from 10-day-old plate cultures and adjusted to 1 × 10^5^ spore mL^−1^ using a hemocytometer under an optical microscope. For each assay, 900 µL of spore suspension and 100 µL of the test solution were thoroughly mixed. A 10–20 µL droplet of this mixture was then placed on the surface of microscopic slides and incubated in a plastic plate with appropriate humidity (80%) at 25 °C. Spore germination was evaluated for approximately 100 spores per treatment under the microscope, and the inhibition rates were calculated based on the germination rates. Each test was performed at least three independent replicates.

For appressorium formation assays, appressorium formation was measured on hydrophobic coverslips (12545100, Fisherbrand, Pittsburgh, PA, USA) at 25 °C for 8 h and 24 h, as described previously [32]. The rate of appressorium formation was observed and calculated by microscopic examination. Three replicates were performed for each treatment and control group.

### 4.5. Scanning Electron Microscopy (SEM) Observations

To investigate the impact of ACEO on the microstructure of *M. oryzae*, hyphae were observed using SEM according to the previously reported methods [44]. Mycelia blocks (1 cm × 0.3 cm × 0.3 cm) were collected after treatment with 5 mg/mL of EO and fixed with 2.5% glutaraldehyde at 4 °C overnight. The fixed specimens were then processed according to the following procedure: First, the samples were washed three times with 0.1 M phosphate-buffered saline (PBS, pH 7.0) for 15 min. Then the samples were postfixed with 1% osmic acid solution for 2 h. After another three washes with PBS, the samples were dehydrated with 50%, 70%, 80%, 90%, and 95% ethanol solution for 15 min at each concentration, and then treated with 100% ethanol for 20 min. Next, the samples were transferred to a 1:1 (*v*/*v*) mixture of ethanol and isoamyl acetate for 30 min, followed by immersion in pure isoamyl acetate for 1 h. Finally, the samples were dried in a Hitachi HCP-2 critical point dryer and coated with a conductive layer. Observations were carried out under a TM-1000 scanning electron microscope (Hitachi, Tokyo, Japan).

### 4.6. Transmission Electron Microscopy (TEM) Observations

To examine the effects of ACEO on *M. oryzae* ultrastructure, the TEM observations were performed according to the previously reported methods [45]. The mycelia treated with EO were fixed in 2.5% glutaraldehyde solution overnight at 4 °C. Subsequently, the samples were processed for TEM as follows: the samples were washed three times with 0.1 M PBS (pH 7.0) and then fixed with 1% osmic acid for 2 h. Next, the mycelia were dehydrated through a graded series of ethanol, followed by acetone. Thereafter, the samples were infiltrated sequentially with a mixture of embedding medium and acetone (*v*/*v* = 1/1) for 1 h, a mixture (*v*/*v* = 3/1) for 3 h, and then with pure embedding medium overnight. The processed samples were sectioned using a Reichert ultramicrotome to a thickness of 70–90 nm. Sections were then stained with lead citrate and uranyl acetate (saturated solution in 50% ethanol), each for 15 min. Finally, observations were conducted using an H-7650 transmission electron microscope (Hitachi, Tokyo, Japan).

### 4.7. Pathogenicity Tests

2-week-old ‘CO39’ rice seedlings and 7-day-old ‘Golden Promise’ barley seedlings were used for inoculation assays. Detached leaf sections (6 cm in length) were placed on two layers of moistened filter paper within 9 cm Petri dishes. The leaf ends were secured with moist cotton wool or filter paper. A conidial suspension (1–2 × 10^5^ conidia/mL), containing different concentrations of ACEO diluted in 0.025% Tween-20, was then spot-inoculated onto the leaves. Each leaf was inoculated with three evenly spaced droplets. Following inoculation, the samples were initially incubated under dark, humid conditions for 24 h. Subsequently, they were transferred to a controlled-environment room set at 25 °C with a 12 h light/dark photoperiod and 90% relative humidity. Disease lesions were examined and photographed seven days post-inoculation. The lesion areas were then quantified using ImageJ version 1.53 software.

### 4.8. GC-MS Analysis

The chemical composition of EO was determined using GC-MS according to the methodology described by Mrabti [46]. Separation was performed on an HP-5MS quartz capillary column (30 m × 0.25 mm, 0.25 μm film thickness) with the following temperature program: initial hold at 40 °C for 1 min, increased to 180 °C at 5 °C/min and held for 2 min, then raised to 280 °C at 10 °C/min, and finally maintained until the end of the analysis. Helium was used as the carrier gas at a constant flow rate of 1.0 mL/min. The sample (1 µL) was injected in splitless mode with the injector temperature set at 250 °C. The mass spectrometer was operated in electron ionization (EI) mode at 70 eV, with the ion source and interface temperatures maintained at 200 °C and 250 °C, respectively. Mass spectra were acquired across the range of 40–600 amu. Quantitative analysis of the components was carried out by the area normalization method based on the chromatographic peak areas.

### 4.9. Data Analysis

Statistical analysis was performed by SPSS version 26.0 software with a one-way analysis of variance and Tukey’s Honest Significant Difference (HSD) test. Statistical significance between the test and control samples was defined as *p* < 0.05.

## 5. Conclusions

This study demonstrates that ACEO possesses potent antifungal activity against *M. oryzae*, with the EC_50_ value of 0.37 µL/mL. Moreover, ACEO severely impaired infection-related processes in *M. oryzae*, such as conidial germination and appressorium development, and caused extensive ultrastructural damage to hyphae. Furthermore, the ultrastructural damage observed, including hyphal wrinkling, cell wall thinning, organelle dissolution, and vacuolation, provides direct cellular evidence for its fungicidal action. These effects collectively resulted in the reduction in fungal pathogenicity, supporting ACEO’s potential as an eco-friendly agent for managing this disease. Further research is needed to identify its active constituents and elucidate their molecular mechanisms of action, which would advance strategies for sustainable rice blast control.

## Figures and Tables

**Table 1:** The major components and their relative contents (%) of ACEO.

No.	Compound	Area (%)
1	m-Xylene	0.45
2	Camphene	0.54
3	E,E-6,11-Tridecadien-1-ol acetate	0.21
4	trans-*β*-Ocimene	0.31
5	Camphor	0.71
6	3-Methyl-2,4,10-trioxatricyclo[3.3.1.1^3,7^]decane	0.29
7	Copaene	0.25
8	*β*-Elemene	1.06
9	Caryophyllene	0.81
10	1,5-dimethyl-8-(1-methylethylidene)-1,5-cyclodecadiene	0.27
11	Calarene	0.64
12	4-trans-propenylveratrole	2.06
13	cis-Isoeugenol methyl ether	1.23
14	Methyl isoeugenol	0.47
15	*γ*-cadinene	0.49
16	Zizanene	0.26
17	Germacrene D	0.28
18	6-Epishyobunone	4.62
19	Ledene	2.78
20	Shyobunone	0.28
21	*α*-Gurjunene	0.92
22	Epishyobunone	8.07
23	*δ*-Cadinene	4.52
24	Isoshyobunone	14.92
25	(Z)-Methylisoeugenol	0.75
26	*α*-Calacorene	4.81
27	*β*-Calacorene	0.78
28	Eremophila ketone	0.30
29	Spathulenol	0.24
30	*β*-Asarone	19.83
31	*α*-Asarone	4.03
32	Dehydroxy-isocalamendiol	2.98
33	*τ*-Cadinol	0.90
34	*α*-Cadinol	0.76
35	3−Chloro−2−fluoro−N−(2−phenylethyl)−N−heptylbenzamide	0.38
36	cis-Calamenene	0.85
37	1,2,3,4-Tetrahydro-3-isopropyl-5-methyl-1-oxonaphthalene	1.26
38	Zierone	8.30
39	Nootkatone	0.39
40	Isocalamenediol	2.99
41	Dibutyl phthalate	0.25
42	Hexamethylcyclotrisiloxane	0.22
43	2-(Acetoxymethyl)-3-(methoxycarbonyl)biphenylene	0.63

Area (%) = Peak volume percentage of compounds.

## Data Availability

The original contributions presented in this study are included in the article. Further inquiries can be directed to the corresponding authors.

## Abbreviations

The following abbreviations are used in this manuscript:

EO	Essential oil
ACEO	*Acorus calamus* Essential oil
GC-MS	Gas chromatography–mass spectrometry
CM	Complete Medium
SEM	Scanning electron microscopy
TEM	Transmission electron microscopy
EC_50_	Median effective concentration
PBS	Phosphate-buffered saline
